# New ethical challenges of digital technologies, machine learning and artificial intelligence in public health: a call for papers

**DOI:** 10.2471/BLT.18.227686

**Published:** 2019-01-01

**Authors:** Diana Zandi, Andreas Reis, Effy Vayena, Kenneth Goodman

**Affiliations:** a Service Delivery and Safety, World Health Organization, avenue Appia 20, 1211 Geneva 27, Switzerland.; b Information, Evidence and Research, World Health Organization, Geneva, Switzerland.; c Institute of Translational Medicine, Eidgenössische Technische Hochschule Zürich, Zürich, Switzerland.; d Institute for Bioethics and Health Policy, University of Miami, Miami, United States of America.

Digital technologies and their applications to public health^1^ are expanding quickly. The World Organization’s (WHO’s) Member States are increasingly adopting the use of digital technologies in the health sector, exploring the use of data for decision-making and considering new solutions to strengthen their health systems.^1^

Access to wellness apps has expanded considerably and people are increasingly searching the internet for health-related information. Through digital health tools and applications, individuals have been offered an opportunity to manage their own health, connect to their health providers and contribute to public health campaigns. Using these new opportunities, a large amount of health data is being constantly generated by users – and captured by service providers. These data are being described as an asset to transform health care and global public health.^2^ By improving the ability to gather, analyse, manage and exchange real-time data and information, digital technologies improve timeliness and accuracy of decision-making and facilitate disease monitoring and surveillance.^3^

Artificial intelligence is the use of coded computer software routines with specific instructions to perform tasks for which a human brain is normally considered necessary. Such tasks include understanding and processing language, recognizing sounds, identifying objects and learning patterns to perform problem-solving operations. The coded software rules are called algorithms. Machine learning is a way of continuously refining an algorithm. The refinement process involves the use of large amounts of data and it is done automatically allowing the algorithm to change with the aim of improving the precision of the artificial intelligence. These technologies promise great benefits to the practice of medicine and to the health of populations.^4^ This is especially true in epidemiology and the tracking of outbreaks of infectious diseases, behavioural science, precision medicine and the modelling and treatment of rare and/or chronic diseases.^5^

At the same time, the collection, storage, use and sharing of large data sets pose many ethical questions regarding governance, quality, safety, standards, privacy and data ownership. Do we know enough about the algorithms that are used to generate the outputs? Are these algorithms adapted for high-, low- and middle-income countries? What safeguards need to be in place as scientists develop and advance the use of artificial intelligence in the fields of clinical and public health? How can the global community ensure that low- and middle-income countries are as able to reap the benefits of these developments as high-income countries? What are the limitations, level of inclusiveness and quality of data sets used for self-learning artificial intelligence algorithms? What can we learn from the use of artificial intelligence in other areas of public policy that would help us navigate the pitfalls associated with this new technology?

The *Bulletin of the World Health Organization* will publish a theme issue on new ethical challenges of digital technologies, machine learning and artificial intelligence in public health. This special issue aims to explore and highlight potential ethical and governance matters that artificial intelligence applications are raising in public health.

We welcome papers covering good research practices, implementation challenges, key normative questions and analysis of ethical challenges that arise in countries dealing with governance, research and implementation of such digital technologies for health. 

The deadline for submission is 15 May 2019. Manuscripts should be submitted in accordance with the *Bulletin’s* guidelines for contributors (available at: http://www.who.int/bulletin/volumes/96/1/18-990118/en/) and the cover letter should mention this call for papers.
